# Natural terpenoids with therapeutic potential against pulmonary arterial hypertension

**DOI:** 10.3389/fphar.2025.1713745

**Published:** 2026-01-22

**Authors:** Kaijie Dang, Junru Zhang, Kexin Yu, Xinyu Liu, Yi Zhu, Xiuli Yang, Xiao Liu, Chuantao Zhang

**Affiliations:** 1 Department of Respiratory Medicine, Hospital of Chengdu, University of Traditional Chinese Medicine, Chengdu, China; 2 Department of Respiratory and Critical Care Medicine, Affiliated Fifth People’s Hospital of Chengdu University of Traditional Chinese Medicine, Chengdu, China

**Keywords:** molecular mechanism, natural products, pharmacologicaleffects, pulmonary arterial hypertension, terpenoids

## Abstract

Pulmonary arterial hypertension (PAH) is a devastating disorder characterized by progressive pulmonary vascular remodeling, chronic inflammation, and right heart failure. Current vasodilation-focused therapeutic strategies often fail to reverse established vascular structural lesions and inadequately control disease progression. Natural terpenoids demonstrate significant anti-remodeling potential through their structural diversity and multi-target intervention capabilities, including suppression of oxidative stress, blockade of inflammatory pathways, regulation of endothelial-mesenchymal transition (EndMT), restoration of the cellular proliferation/apoptosis balance, and recovery of ion channel functionality. This review systematically summarizes the pharmacological effects, molecular mechanisms, and limitations of natural terpenoids in ameliorating PAH, providing novel perspectives for targeted therapies. To advance clinical translation, future efforts must prioritize large-scale trials validating safety profiles and optimizing dosing regimens, while diversifying natural sources to accelerate precision drug development.

## 1 Introduction

Pulmonary arterial hypertension (PAH) is a progressive and highly lethal cardiopulmonary disorder characterized by sustained elevation of pulmonary arterial pressure (PAP) and pulmonary vascular resistance (PVR), primarily resulting from abnormal pulmonary vascular remodeling and excessive proliferation of pulmonary arterial smooth muscle cells (PASMCs) (Zhang et al., 2024a). This pathological process subsequently leads to right ventricular hypertrophy (RVH), RV diastolic and systolic dysfunction, and ultimately right heart failure, which severely threatens patient survival (Yu Z. et al., 2022). Epidemiological data indicate that despite advances in clinical diagnosis and treatment, the long-term prognosis of PAH remains poor, with a 5-year mortality rate exceeding 40% in many patient cohorts (Langleben et al., 2022).

Currently, the mainstay of PAH management relies on pharmacological interventions such as endothelin receptor antagonists, phosphodiesterase-5 inhibitors, and prostacyclin analogs, which primarily target vasodilation (Langleben et al., 2022; Zhang et al., 2024b). However, these drugs offer only symptomatic relief, without fundamentally reversing the underlying vascular pathological remodeling or halting disease progression. Moreover, their long-term administration is frequently associated with adverse effects, high treatment costs, and limited accessibility in low-resource settings. Consequently, the development of safer, more effective, and mechanism-oriented therapeutic strategies has become an urgent focus in the field of PAH research.

Natural terpenoids, a diverse class of secondary metabolites widely distributed in plants, possess a broad spectrum of pharmacological properties, including antioxidant, anti-inflammatory, vasodilatory, and cardiovascular protective effects (Oliveira et al., 2021; Abdul Ghani et al., 2023). Recent experimental studies have highlighted their potential role in PAH prevention and treatment through multiple mechanisms, such as inhibiting pulmonary vascular remodeling, suppressing excessive PASMCs proliferation, reducing oxidative stress, and modulating apoptosis (Silva et al., 2021). Notably, certain monoterpenes have demonstrated the ability not only to lower pulmonary arterial pressure but also to improve endothelial function via regulation of ion channel activity, thereby reducing PVR and alleviating the hemodynamic burden in PAH (Silva et al., 2021; Zeng et al., 2023).

Emerging preclinical evidence further supports this therapeutic potential: for instance, 4-terpineol has been shown to mitigate hypoxia-induced pulmonary arterial remodeling in PAH rat models (Gu et al., 2023), while perillyl alcohol administration has produced favorable hemodynamic improvements in experimental PAH (Beik et al., 2021). At the molecular level, terpenoids may suppress abnormal PASMC proliferation by regulating mitochondrial function, ion channel dynamics, and specific signaling pathways, such as downregulating the 12-lipoxygenase/12-hydroxyeicosatetraenoic acid axis, thereby significantly reducing PVR and improving hemodynamic parameters (Zhang C. et al., 2018; Ruffenach et al., 2024; Wang et al., 2025).

In summary, accumulating evidence suggests that natural terpenoids not only have the potential to serve as adjuvant therapies complementing existing pharmacological regimens but may also emerge as promising frontline agents for early intervention in PAH, offering the dual advantages of minimizing adverse effects and improving long-term prognosis (Oliveira et al., 2021). Based on this rationale, the present review aims to comprehensively summarize recent advances in terpenoid-based research for PAH prevention and treatment, with a particular emphasis on their underlying mechanisms of action, targeted signaling pathways, and translational potential, thereby providing a robust theoretical framework and scientific basis for the development of novel, personalized therapeutic strategies in PAH.

## 2 Methods

The literature for this review was retrieved from PubMed, ScienceDirect, Web of Science, and Google Scholar databases, covering publications up to September 2025 and restricted to English-language sources. The search strategy employed the terms “pulmonary arterial hypertension,” “natural products,” “terpenoids,” and “terpenes,” as well as various combinations of these keywords. Approximately 160 relevant publications, comprising both original research and reviews, were selected for this analysis.

## 3 Classification and pharmacological activities of natural terpenoids

### 3.1 Classification of natural terpenoids

**FIGURE 1:**
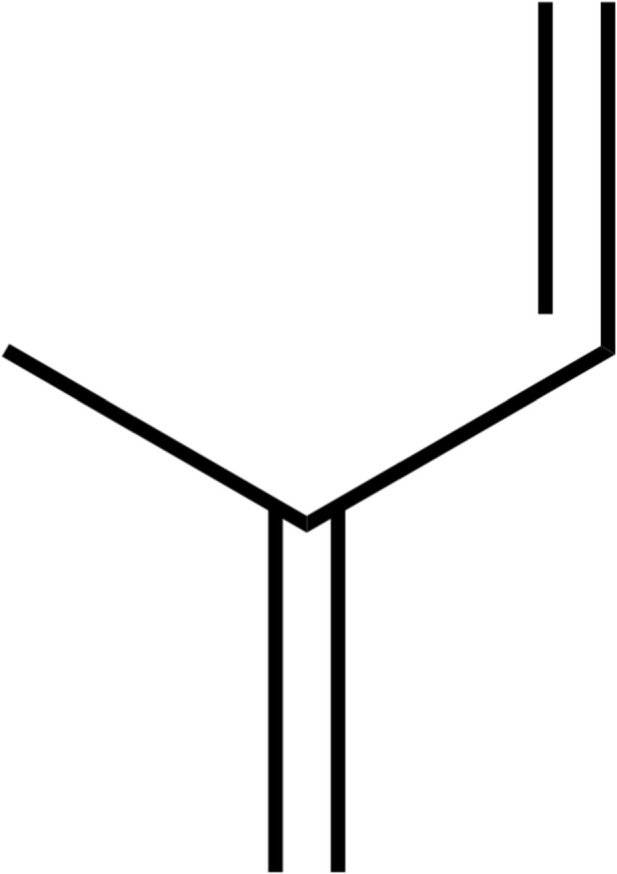
Chemical structural formula of isoprene.

**FIGURE 2:**
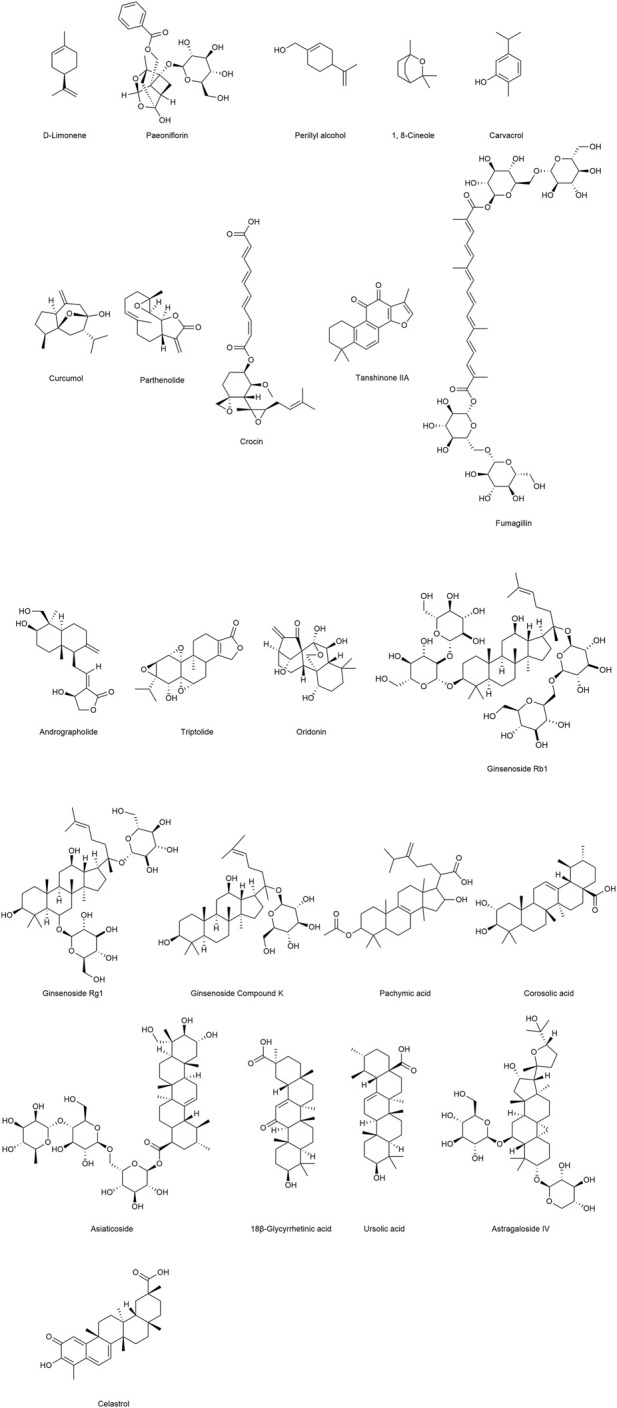
Representative natural terpenoid compounds for the treatment of PAH.

Terpenoids constitute a vast and diverse class of naturally occurring organic compounds, ubiquitously distributed throughout the plant kingdom (Johnson et al., 2023; Hoshino and Villanueva, 2023). Their fundamental structural framework is built upon the isoprene unit, a five-carbon building block, as depicted in Figure 1 (El-Baba et al., 2021). As integral products of plant secondary metabolism, terpenoids are especially abundant in the essential oils of aromatic plants, where they contribute not only to fragrance but also to ecological interactions and defense mechanisms (Mathur et al., 2024). Depending on the number of isoprene units incorporated into their carbon skeleton, these compounds are classified into distinct subclasses, including monoterpenoids, sesquiterpenoids, diterpenoids, triterpenoids, and tetraterpenoids (Burger et al., 2021). In the present article, we provide a systematic overview of naturally derived terpenoids with potential applications in the prevention and treatment of pulmonary arterial hypertension (PAH), with representative molecular structures illustrated in Figure 2.

### 3.2 Pharmacological activities of natural terpenoids

As structurally diverse and functionally versatile secondary metabolites of plants, natural terpenoids have attracted considerable attention for their pharmacological potential in the prevention and treatment of cardiovascular diseases, particularly PAH. Accumulating evidence demonstrates that terpenoids exert a broad spectrum of bioactivities, including potent anti-inflammatory and antioxidant effects, suppression of vascular remodeling, and regulation of vascular smooth muscle cell and endothelial cell proliferation and apoptosis. Moreover, they can inhibit the persistent activation of multiple aberrant signaling pathways associated with PAH pathogenesis. Certain terpenoids are also capable of attenuating endothelial-to-mesenchymal transition (EndMT), thereby contributing to the preservation of vascular structural integrity and the restoration of endothelial function. Through these pharmacological actions, terpenoids provide multi-target and multi-pathway therapeutic strategies aimed at delaying, or potentially reversing, the pathological progression of PAH. In this review, we compile and critically assess 23 representative natural terpenoids reported to be effective in PAH prevention and treatment. Their botanical sources, molecular targets, principal signaling pathways, and major pharmacological effects are systematically summarized and comparatively analyzed, with detailed information presented in Table 1.

**TABLE 1:** Mechanisms of natural terpenoids in the treatment of PAH.

Natural terpenoid	Research model	Concentration	Mechanisms	Targets/Pathways	References
D-Limonene	*In vivo*: MCT-induced rats	300 mg/kg/day	↓inflammation	↓IL-1β, IL-6, TNF-α; ↑IL-10	Teixeira-Fonseca et al. (2024)
Paeoniflorin	*In vivo*: male SD rats induced by chronic hypoxia and SU5416 *In vitro*: human PAECs	300 mg/kg/day 10 μM	↓EndMT	↓fibronectin, vimentin, α-SMA, Snail, Twist; ↑VE-cadherin, BMPR2	Yu et al. (2020)
	*In vitro*: hypoxia-induced PASMCs from male SD adult rats	0.02, 0.2, 2, 20 μmol/L	↓proliferation	↑A2BAR; ↓S-phase cell proportion	Qian et al. (2013)
	*In vivo*: MCT-induced male adult SD rats *In vitro*: human PAECs	100, 200, 300 mg/kg/day 10 μM	↓EndMT ↓inflammation ↑apoptosis	↓CD31, α-SMA, vimentin↑VE-cadherin, BMPR2 ↓CD68+, IL-6, IL-1β, TNF-α, tryptase, TGFβ1, p-TAK1, p-p38 MAPK, p-ERK1/2, p-p65 NF-κB	Yu et al. (2022a)
Perillyl alcohol	*In vivo*: MCT-induced male Wistar rats	20, 30, 40, 50, 60 mg/kg/day	↓inflammation ↑apoptosis ↓oxidative stress	↓TNF-α, IL-6 ↑Bax, p21; ↓Bcl-2 ↑SOD, GPx, CAT, TAC	Beik et al. (2021)
	*In vivo*: MCT-induced male Wistar rats	50 mg/kg/day	↓inflammation ↑apoptosis	↓IL-1β, IL-8 ↓PARP1, HIF1α, NFATc2, α-SMA; ↑miR-204	Rajabi et al. (2020)
	*In vivo*: MCT-induced male Wistar rats	50 mg/kg/day	↓inflammation ↓oxidative stress ↑apoptosis	↓IL-6, TNF-α ↑TAC; ↓MDA ↓Bax, Caspase-3; ↑Bcl-2, miR-204	Rajabi et al. (2021)
1,8-Cineole	*In vivo*: MCT-induced male Wistar rats *In vitro*: highpressure-induced H9c2 and NRVMs	25 mg/kg/day 50–800 μg/mL	↑Ca^2+^ homeostasis ↑electrical conductivity ↑autophagy ↑mitochondrial homeostasis	↑SERCA2a, RyR2 ↑Cx43 ↑LC3-II, Parkin ↓p-Drp1; ↑MFN2, TFAM	Alves-Silva et al. (2022)
	*In vivo*: MCT-induced male Wistar rats *In vitro*: hypoxia-induced human PAECs	25, 100 mg/kg/day 400 μg/mL	↓EndMT ↓proliferation ↑gap junction	↑VE-cadherin, ZO-1, BMPR2, p-Smad1/5, PPAR-γ; ↓SMURF1 ↓MMP-9 ↑Cx43	Alves-Silva et al. (2025)
Carvacrol	*In vivo*: hypoxia-induced male Wistar rats *In vitro*: hypoxia-induced rats PASMCs	25, 50, 100 mg/kg 200–800 μM	↑apoptosis ↓oxidative stress	↓ΔΨm, Bcl-2, procaspase-3, p-ERK1/2, p-Akt; ↑caspase-3 ↓MDA; ↑SOD, GSH	Zhang et al. (2016)
Curcumol	*In vivo*: MCT-induced male SD rats *In vitro*: TNF-α, TGF-β1 and IL-1β induced rat PAECs	12.5, 25, 50 mg/kg/day 50, 100 mg/L	↓EndMT ↓inflammation	↑CD31, vWF; ↓α-SMA, vimentin, Snail, p-AKT, p-GSK3β ↓IL-6, IL-1β, TNF-α	Nie et al. (2023)
Parthenolide	*In vivo*: hypoxia-induced male SD rats *In vitro*: hypoxia-induced rats PASMCs	10, 30 mg/kg/every other day 1–16 μM	↓proliferation ↑apoptosis	↓PCNA, CyclinD1, p-STAT3 ↑Annexin V, Bax/Bcl-2; ↓p-STAT3	Yao et al. (2024)
Fumagillin	*In vivo*: MCT-induced male SD rats *In vitro*: RPASMCs	0.5 mg/kg/every other day 20 nM	↓inflammation ↓proliferation ↑apoptosis	↓CD45+ ↓MetAP2	Kass et al. (2012)
Tanshinone IIA	*In vivo*: hypoxia-induced male SD rats *In vitro*: hypoxia-induced rats PASMCs	10 mg/kg/day 0–50 μg/mL	↓proliferation	↑cells in G1/G0-phase, p27; ↓p-AKT, Skp2	Luo et al. (2013)
	*In vitro*: hypoxia-induced male SD rats	0.01–600 μM	↑Ca^2+^ homeostasis	↓extracellular Ca^2+^ influx, intracellular Ca^2+^ release; ↑KCa channel	Wang et al. (2010)
	*In vivo*: MCT-induced male SD rats	10 mg/kg/day	↑vasoactive factor	↑p-PI3K, p-Akt, p-eNOS	Zhang et al. (2022a)
	*In vivo*: hypoxia-induced male SD rats	10 mg/kg/day	↑voltage-gated potassium ion channels	↑KV1.5, KV2.1, IKV; ↓Ca^2+^ influx	Zheng et al. (2015)
Crocin	*In vivo*: MCT-induced male SD rats	7.5, 15, 30 mg/kg/day	↓oxidative stress	↑SOD, CAT, GPx, GSH, TAC, OXR1, p21, Nrf2; ↓MDA	Dianat et al. (2020)
	*In vivo*: hypoxia-induced male C57BL/6 mice *In vitro*: PDGF-BB or TGF-β1 induced mice PAFs	50 mg/kg/every 3 days 10, 50 μM	↓inflammation ↓proliferation	↓ECM ↓α-SMA, Col1a1, MMP2, TGF-β1, p-Smad3; ↑TIMP1	Deng et al. (2024)
	*In vivo*: MCT-induced male SD rats	200 mg/kg/day	↓inflammation ↓oxidative stress ↓proliferation	↓CCL2, CCR2, TNF-α, IL-6, IL-1β, ↓MDA; ↑SOD, GSH-Px, iNOS ↓α-SMA	Sheng et al. (2022)
Andrographolide	*In vivo*: hypoxia-induced mice and Sugen5416-induced mice *In vitro*: PH-PASMCs	1 mg/kg/day 1–100 μM	↓inflammation ↓oxidative stress ↑Ca^2+^ homeostasis ↓proliferation ↓vasoactive factor	↓TLR4, NF-κB p65, IL-6, IL-8 ↓ROS, NOX2, NOX4, p47phox, MDA, 4-HNE; ↑SOD,Nrf2, HO-1 ↓[Ca^2+^]i ↓ERK, JNK; ↑p38, BMPR2 ↓ET-1, VEGF	Nie et al. (2021)
Triptolide	*In vivo*: MCT-induced male SD rats	0.20, 0.25 mg/kg/every other day	↓proliferation	↓MMP2, MMP9	Wei et al. (2007b)
Oridonin	*In vivo*: hypoxic-hypercapnia-induced male SD rats	10 mg/kg/day	↓proliferation ↓oxidative stress	↓PCNA ↑SOD; ↓MDA	Wang et al. (2009)
Ginsenoside Rb1	*In vitro*: male SD rats induced by hypoxia and MCT	0.1–300 μM	↑Ca^2+^ homeostasis	↓SOCE, Mn2+ influx, [Ca2+]i	Wang et al. (2015a)
	*In vivo*: MCT-induced male SD rats	30 mg/kg/day	↑Ca^2+^ homeostasis	↓SOCE, [Ca2+]I, STIM2, TRPC1, TRPC4	Wang et al. (2020a)
Ginsenoside Rg1	*In vivo*: hypoxia-induced C57BL/6 and Capn1-KO mice *In vitro*: hypoxia-induced rats PASMCs	5, 10, 20 mg/kg/day 20 μM	↓proliferation ↓inflammation ↓fibrosis	↓PCNA, Ki-67,calpain-1, p-STAT3 ↓IL-6, TNF-α, VCAM-1, ICAM-1 ↓α-SMA, TGF-β1, Collagen I, HYP	Ran et al. (2024)
	*In vivo*: hypoxia-induced male SD rats *In vitro*: hypoxia-induced human PAECs	10, 20 mg/kg/day 20 μM	↓inflammation ↓EndMT	↓TNF-α, IL-1β, p-NF-κB p65; ↑CCN1 ↑CD31, VE-cadherin; ↓α-SMA, Vimentin, TGF-β1, p-Smad2/3	Tang et al. (2023a)
	*In vivo*: rats induced by Sugen5416 and hypoxia	10, 15, 20 mg/kg/day	↓inflammation ↓apoptosis	↓NF-κB, IL-6, IL-8, cGAS, STING, p-STING, TBK1, p-TBK1 ↓p16, p21	Ding et al. (2025)
Ginsenoside Compound K	*In vitro*: rats PASMCs induced by PDGF-BB	1, 3, 5 μmol/L	↓proliferation	↓S-phase cell proportion, cyclinD1, β-catenin; ↑α-SMA, SM22α, pGSK-3β/GSK-3β	Liu et al. (2021)
Pachymic Acid	*In vivo*: hypoxia-induced male SD rats *In vitro*: hypoxia-induced rats PASMCs	5 mg/kg/day	↓oxidative stress ↓proliferation	↓ROS, MDA, Keap1; ↑SOD, GSH-Px, Nrf2, HO-1, SOD-1	He et al. (2022)
Corosolic acid	*In vivo*: MCT-induced male SD rats *In vitro*: PASMCs from normal subjects and IPAH patients	1 mg/kg/day 0.1–30 μM	↓proliferation	↓STAT3	Kawade et al. (2023)
	*In vivo*: MCT-induced male SD rats *In vitro*: PASMCs from normal subjects and IPAH patients	1 mg/kg/day 100 mM	↓proliferation ↓inflammation	↓PDGFRβ, STAT3 ↓NF-κB, M1 and M2 macrophages	Yamamura et al. (2024)
Asiaticoside	*In vivo*: hypoxia-induced male SD rats *In vitro*: hypoxia-induced human PAECs	50 mg/kg/day 50 μg/mL	↓apoptosis ↓vasoactive factor ↓inflammation	↓Caspase-3; ↑p-Akt, p-eNOS ↓ET-1; ↑NO, cGMP	Wang et al. (2018a)
	*In vivo*: hypoxia-induced male SD rats *In vitro*: hypoxia-induced rats PASMCs	50 mg/kg/day 0–200 μg/mL	↓proliferation ↑apoptosis	↓TGF-βRII mRNA, TGF-β1, p-Smad2/3	Wang et al. (2015b)
18β-glycyrrhetinic acid	*In vivo*: SD rats induced by simulated high altitude environment	25, 50, 100 mg/kg/day	↓oxidative stress ↑metabolic pathway	↓MDA; ↑SOD, GSH-Px	Yang et al. (2021b)
	*In vivo*: MCT-induced male SD rats *In vitro*: huanm PASMCs	25, 50, 100 mg/kg/day 20–160 μM	↓proliferation ↑apoptosis	↑cells in G1/G0-phase; ↓RhoA, ROCK1, ROCK2, p-MYPT1/t-MYPT1 ↑Bax, p27kip1; ↓Bcl-2, Bcl-2/Bax	Zhang et al. (2019a)
	*In vivo*: MCT-induced male SD rats *In vitro*: human PASMCs	25, 50, 100 mg/kg/day 0–90 μM	↓inflammation ↓endoplasmic reticulum stress ↓proliferation	↓TNF-α, IL-6, MCP-1, p-NF-κB p65; ↑IκB ↓GRP78, p-PERK, p-eIF2α	Wang et al. (2022b)
	*In vivo*: MCT-induced male SD rats *In vitro*: hypoxia-induced rats PASMCs	100, 200 mg/kg/day 10, 20, 40 μM	↓inflammation ↓oxidative stress ↓proliferation ↑apoptosis	↓IL-1β, NF-κB, IL-6, TNF-α; ↑PPAR-γ ↑SOD, GSH, CAT, iNOS, NO, L-Arg; ↓Vanin-1 ↓α-SMA; ↑cells in G2/M-phase	Shanahati et al. (2025)
Ursolic Acid	*In vivo*: MCT-induced male SD rats *In vitro*: neonatal rat ventricular myocytes	50 mg/kg/day 10 μM	↓fibrosis ↓apoptosis ↑fatty acid metabolism	↓Col1a1, Col3a1, TGF-β1 ↓Bax ↑PPARα, CPT1b	Gao et al. (2020)
Astragaloside IV	*In vivo*: hypoxia-induced C57BL/6 mice *In vitro*: hypoxia-induced mice CD4+T cell hypoxia-induced mice PASMCs	20, 40, 80 mg/kg/day 10, 20 or 40 μM 10, 20 or 40 μM	↓proliferation ↑Immune modulation	↓RhoA; ↑p27 ↓p-mTOR, IL-21, Bcl-6, Tfh cell proportion; ↑Tfr cell proportion, TGF-β, IL-10, CTLA-4	Li C. et al. (2022)
	*In vivo*: hypoxia-induced male SD rats *In vitro*: hypoxia-induced rats PASMCs	2 mg/kg/day 5, 10, 20 μmol/L	↓proliferation	↑cells in G1/G0-phase; ↓Jagged-1, Notch-3, Hes-5	Yao et al. (2021)
	*In vivo*: MCT-induced male SD rats *In vitro*: MCTP-induced human PAECs	40, 80 mg/kg/day 50, 100 μmol/L	↓inflammation	↓IL-18, IL-1β, NLRP-3, ASC, caspase-1, calpain-1	Sun et al. (2021)
	*In vivo*: MCT-induced male SD rats *In vitro*: hypoxia-induced human PASMCs and human PAECs	10, 30 mg/kg/day 10–80 μM	↓proliferation ↑apoptosis ↓inflammation ↑vasoactive factor	↓α-SMA, PCNA, HIF-1α, p-ERK1/2; ↑p27, p21 ↑Bax, cleaved caspase-9, cleaved caspase-3; ↓Bcl-2 ↓TNF-α, IL-1β ↓VEGF	Jin et al. (2021)
Celastrol	*In vivo*: hypoxia-induced male SD rats *In vitro*: hypoxia-induced human PASMCs	0.5, 1 mg/kg/day 0.125–2 μM	↓proliferation ↑apoptosis ↓fibrosis	↓PDE5; ↑PKG, cGMP	Tan et al. (2025)

#### 3.2.1 Monoterpenoids

##### 3.2.1.1 D-Limonene

D-Limonene is a naturally occurring monocyclic monoterpene predominantly present in the peels and essential oils of *Citrus sinensis* (L.) Osbeck (Fam. Rutaceae) (Anandakumar et al., 2021; Rakoczy et al., 2025). The pathological progression of PAH is intimately linked to sustained inflammatory responses and oxidative stress, both of which drive pulmonary vascular remodeling and ultimately result in right ventricular (RV) dysfunction (Igarashi-Sugimoto et al., 2025). Experimental studies have demonstrated that D-limonene exerts potent anti-inflammatory and antioxidant effects—pharmacological properties of particular relevance to the vascular pathological microenvironment of PAH (Hou et al., 2022; Teixeira-Fonseca et al., 2024). At the molecular level, D-limonene downregulates the mitogen-activated protein kinase (MAPK) signaling pathway and the expression of the pro-apoptotic gene B-cell lymphoma 2-associated X protein (Bax), while upregulating the anti-apoptotic gene B-cell lymphoma 2 (Bcl-2), thereby suppressing cardiomyocyte apoptosis (Wang et al., 2018b; Younis, 2020). Such activity may mitigate PAH-induced cardiomyocyte loss and provide indirect protection for RV function, considering that right heart failure remains one of the most common and fatal outcomes of advanced PAH. In addition, D-limonene has been shown to attenuate ischemia-related vascular injury by inhibiting pathological vascular remodeling and enhancing antioxidant defenses, thereby partially restoring vascular homeostasis under PAH conditions (Wang et al., 2018b; Anandakumar et al., 2021).

##### 3.2.1.2 Paeoniflorin

Paeoniflorin is a monoterpene glycoside primarily isolated from the dried roots of *Paeonia lactiflora* Pall*.* (Paeoniaceae) (Li et al., 2023). In rat models of PAH, paeoniflorin has been shown to mitigate pulmonary tissue injury, alleviate PAH symptoms, and markedly improve survival rates by reversing EndMT associated with bone morphogenetic protein receptor type II (BMPR2) downregulation (Yu et al., 2020; Cao et al., 2025). Subsequent investigations have demonstrated that paeoniflorin significantly decreases the levels of multiple fibrosis-related markers in lung tissue, including collagen type IV, α-smooth muscle actin (α-SMA), hyaluronic acid, laminin, and procollagen type III (Yu M. et al., 2022). Moreover, it suppresses the expression of collagen types I and III, thereby effectively attenuating pulmonary vascular remodeling. These anti-fibrotic actions, together with its ability to inhibit inflammatory responses, underscore the potential of paeoniflorin as a multi-target therapeutic agent for delaying or halting the pathological progression of PAH (Yu et al., 2021).

##### 3.2.1.3 Perillyl alcohol

Perillyl alcohol is a naturally occurring monoterpene alcohol widely distributed in the essential oils of various plants, including *Perilla frutescens* (L.) Britton, *Lavandula angustifolia* Mill., and *Mentha* × *piperita* L. (Fam. Lamiaceae) (Zhang L. et al., 2024). Derived primarily from plant volatile oils, it is characterized by a distinctive aromatic fragrance (Chen et al., 2021). Evidence from experimental animal studies demonstrates that perillyl alcohol can dose-dependently and significantly ameliorate the symptoms of PAH by reducing pulmonary arterial pressure, attenuating RVH, and inhibiting pulmonary vascular remodeling (Rajabi et al., 2020; Beik et al., 2021; Kordestani et al., 2024). At the molecular level, it modulates the expression of multiple microRNAs and inhibits the activity of poly(ADP-ribose) polymerase 1 (PARP1), thereby reducing oxidative stress and suppressing aberrant cell proliferation (Rajabi et al., 2020; Puppala et al., 2022). In addition, perillyl alcohol inhibits farnesyltransferase, which regulates inflammation-related signaling pathways and confers synergistic anti-inflammatory and antioxidant effects (Puppala et al., 2022).

##### 3.2.1.4 1,8-Cineole

1,8-Cineole is a major monoterpene oxide abundantly present in the volatile oils of various aromatic plants, particularly *Eucalyptus globulus* Labill. (Myrtaceae) and *Salvia rosmarinus* Spenn (Lamiaceae; syn. *Rosmarinus officinalis* L.) (Pereira-Gonçalves et al., 2018). In the context of PAH, 1,8-cineole exerts protective effects by modulating the dysregulated BMPR2/Smad1/5 and BMPR2/PPAR-γ signaling pathways, thereby inhibiting pulmonary vascular remodeling (Alves-Silva et al., 2025). These actions result in reduced collagen deposition within lung tissue, suppression of excessive vascular smooth muscle cell proliferation, and attenuation of medial layer thickening in pulmonary arteries (Alves-Silva et al., 2025). With respect to oxidative stress, 1,8-cineole significantly decreases the levels of reactive oxygen species (ROS), malondialdehyde (MDA), and lipid peroxides (LPO), while enhancing the enzymatic activities of superoxide dismutase (SOD), glutathione peroxidase (GSH-Px), and catalase (CAT) (Chen et al., 2023; Liu Z. et al., 2023). In terms of immune and inflammatory regulation, it has been shown to inhibit the release of pro-inflammatory cytokines, including tumor necrosis factor-α (TNF-α), interleukin-1β (IL-1β), IL-4, and IL-5, and to restore Th1/Th2 immune balance (Juergens et al., 2020). Furthermore, 1,8-cineole exhibits antihypertensive activity primarily through the inhibition of calcium (Ca^2+^) channels in vascular smooth muscle cells, leading to reduced vascular tone (Pereira-Gonçalves et al., 2018; Nozohour et al., 2022). In PAH, this vasodilatory effect may directly alleviate pulmonary vasoconstriction, thereby improving hemodynamic status and potentially relieving RV strain (Pereira-Gonçalves et al., 2018; Alves-Silva et al., 2022).

##### 3.2.1.5 Carvacrol

Carvacrol is a phenolic monoterpenoid abundantly distributed in the essential oils of aromatic herbs of the family Lamiaceae, such as *Origanum vulgare* L., *Thymus vulgaris* L., and *Satureja hortensis* L. (Singh et al., 2023). In *ex vivo* rat aortic ring assays, carvacrol has been shown to elicit a pronounced vasorelaxant response, while *in vivo* studies confirm its antihypertensive activity (Testai et al., 2016). In terms of antioxidant and anti-inflammatory properties, carvacrol effectively mitigates oxidative stress, suppresses the release of the pro-inflammatory cytokine IL-1β, and enhances the secretion of the anti-inflammatory cytokine interleukin-10 (IL-10) (Zhang et al., 2016; Khazdair et al., 2024; Koçak et al., 2025). At the molecular level, carvacrol inhibits the MAPK signaling pathway and blocks the activity of the transient receptor potential melastatin 7 (TRPM7) channel, thereby disrupting proliferation-related signaling cascades and attenuating pulmonary vascular remodeling (Cai et al., 2021).

#### 3.2.2 Sesquiterpenoids

##### 3.2.2.1 Curcumol

Curcumol is a bioactive sesquiterpenoid extracted from the essential oil of the rhizomes of *Curcuma phaeocaulis* Valeton (Fam. Zingiberaceae) (Zhai et al., 2024). *In vitro* studies have shown that curcumol effectively inhibits EndMT in primary rat pulmonary artery endothelial cells (PAECs) induced by TNF-α, transforming growth factor-β1 (TGF-β1), and interleukin-1β (IL-1β) (Nie et al., 2023). In a monocrotaline (MCT)-induced rat model of PAH, curcumol attenuated RVH and reduced pulmonary arterial wall thickening in a dose-dependent manner, thereby exerting a significant protective effect against pulmonary vascular remodeling (Nie et al., 2023).

##### 3.2.2.2 Parthenolide

Parthenolide, a sesquiterpene lactone predominantly obtained from *Tanacetum parthenium* (L.) Sch.Bip. (Fam. Asteraceae), has demonstrated notable pharmacological potential in PAH (Freund et al., 2020). *In vitro* studies reveal that parthenolide inhibits hypoxia-induced proliferation and migration of PASMCs while promoting their apoptosis (Yao et al., 2024). Mechanistically, parthenolide directly interacts with signal transducer and activator of transcription 3 (STAT3), markedly reducing its phosphorylation and nuclear translocation, thereby suppressing downstream pro-proliferative signaling cascades (Yao et al., 2024). *In vivo*, administration of parthenolide significantly decreased mean pulmonary arterial pressure (mPAP), attenuated pulmonary vascular remodeling, and reduced collagen deposition, highlighting its potential as a therapeutic agent for slowing or preventing PAH progression (Yao et al., 2024).

##### 3.2.2.3 Fumagillin

Fumagillin is a secondary metabolite produced through the fermentation of the fungus *Aspergillus fumigatus* Fresen (Ingber et al., 1990). Experimental studies have shown that fumagillin markedly reduces the infiltration of CD45^+^ immune cells into lung tissue, thereby alleviating inflammatory responses (Kass et al., 2012). At the molecular level, fumagillin inhibits the enzymatic activity of methionine aminopeptidase-2 (MetAP2), which in turn blocks platelet-derived growth factor (PDGF)-driven proliferation of smooth muscle cells, ultimately suppressing pulmonary vascular remodeling (Kass et al., 2012). Owing to this combination of anti-inflammatory and anti-proliferative effects, fumagillin holds potential as a multi-target therapeutic candidate for the prevention and treatment of PAH.

#### 3.2.3 Diterpenoids

##### 3.2.3.1 Tanshinone IIA

Tanshinone IIA, a major lipophilic diterpene quinone isolated from the phytomedicine *Salvia miltiorrhiza* Bunge (Danshen), is predominantly concentrated in its roots and rhizomes (Guo et al., 2020). Experimental studies have demonstrated that tanshinone IIA can suppress hypoxia-induced pulmonary vascular structural abnormalities, thereby attenuating the severity of PAH (Zhang N. et al., 2018; Li et al., 2022b). Mechanistically, tanshinone IIA inhibits the aberrant proliferation of PASMCs under hypoxic conditions by modulating the Protein kinase B (Akt)/S-phase kinase-associated protein 2 (Skp2)/Cyclin-dependent kinase inhibitor 1B (p27) signaling pathway (Luo et al., 2013). It also reduces the release of pro-inflammatory cytokines, including TNF-α, IL-1β, and interleukin-6 (IL-6), thereby attenuating pulmonary inflammation (Fu et al., 2021; Yang L. et al., 2021). From an antioxidant perspective, tanshinone IIA activates the nuclear factor erythroid 2-related factor 2 (Nrf2) pathway, enhancing the activities of SOD and CAT while reducing lipid peroxide accumulation, thus preventing oxidative stress-induced cellular injury (Gu et al., 2016; Fu et al., 2021; Wu M. et al., 2024). Moreover, tanshinone IIA exerts multi-target vascular protective effects by regulating both Ca^2+^ and K^+^ channel activities, significantly improving vascular dysfunction in hypoxic PAH (Wang et al., 2010). Notably, its water-soluble derivative, sodium tanshinone IIA sulfonate, has been shown in animal studies to inhibit the MAPK signaling pathway, thereby reducing oxidative stress damage and mitigating pulmonary vascular remodeling (Bao et al., 2020; Xu et al., 2025).

##### 3.2.3.2 Crocin

Crocin, a naturally occurring carotenoid glycoside isolated from saffron (*Crocus sativus L*.), exhibits potent anti-inflammatory and antioxidant activities that underpin its diverse pharmacological effects (Sheng et al., 2022; Xi et al., 2025). In terms of inflammation regulation, crocin downregulates the C-C motif chemokine ligand 2/C-C chemokine receptor 2 (CCL2/CCR2) signaling pathway, thereby reducing pulmonary levels of pro-inflammatory cytokines such as TNF-α and IL-6 (Sheng et al., 2022). With respect to oxidative stress, crocin effectively mitigates ROS-induced pulmonary vascular injury, alleviating oxidative burden and preventing further tissue damage (Dianat et al., 2020; Sheng et al., 2022). From a hemodynamic perspective, crocin significantly decreases right ventricular systolic pressure (RVSP) and mPAP, thereby reducing RV afterload. It also inhibits the abnormal proliferation of PASMCs, attenuates pulmonary vascular wall thickening, and suppresses fibrotic processes, effectively reversing pulmonary vascular remodeling (Sheng et al., 2022). Additionally, crocin restores the balance between matrix metalloproteinase-2 (MMP-2) and tissue inhibitor of metalloproteinase-1 (TIMP-1), contributing to the maintenance of pulmonary vascular structural homeostasis (Deng et al., 2024).

##### 3.2.3.3 Andrographolide

Andrographolide, the major bioactive constituent extracted from *Andrographis paniculata* (Burm.f.) Wall. ex Nees (Fam. Acanthaceae), is widely recognized for its therapeutic use in the treatment of inflammation, infections, and cardiovascular diseases (Hsu et al., 2016; Wong et al., 2021). Studies have demonstrated that andrographolide significantly alleviates pulmonary inflammation and oxidative stress associated with PAH by suppressing the expression of pro-inflammatory factors TNF-α, IL-1β, C-X-C motif chemokine ligand 1 (CXCL1), and the oxidative damage marker 8-hydroxy-2′-deoxyguanosine, thereby improving PAH-related pulmonary vascular remodeling (Wu et al., 2017; Tran et al., 2021; Liu B. et al., 2022). Animal studies have further confirmed that this compound markedly reduces collagen deposition and inflammatory cell infiltration in lung tissue (Tan et al., 2016; Karkale et al., 2018). With respect to cardioprotection, andrographolide can improve PAH-induced ventricular remodeling and cardiac dysfunction by blocking the MAPK signaling pathway (Wu et al., 2017; Othman and Mohd Azman, 2022; Tian et al., 2023). Moreover, it modulates the abnormal proliferation and apoptosis of PASMCs and endothelial cells, thus inhibiting pathological vascular structural alterations at a multicellular level (Nie et al., 2021; Han et al., 2023; Shan et al., 2025).

##### 3.2.3.4 Triptolide

Triptolide, a highly bioactive diterpenoid triepoxide isolated from the phytomedicine *Tripterygium wilfordii* Hook.f. (Fam. Celastraceae), possesses potent anti-inflammatory, immunosuppressive, and anti-proliferative properties (Zhang et al., 2020). *In vivo* studies have demonstrated that triptolide markedly alleviates MCT or pneumonectomy-induced PAH and the associated RVH by inhibiting pathological cell proliferation, suppressing inflammatory responses, and improving pulmonary vascular remodeling (Wei et al., 2007a). Moreover, triptolide not only slows PAH progression but can also partially reverse established vascular remodeling by inhibiting the activities of MMP-2 and matrix metalloproteinase-2 (MMP-9), thereby contributing to the downregulation of pulmonary inflammation (Wei et al., 2007b).

##### 3.2.3.5 Oridonin

Oridonin, a bioactive ent-kaurane diterpenoid isolated from the phytomedicine *Isodon rubescens* (Hemsl.) H.Hara (syn. *Rabdosia rubescens*), exhibits a wide range of pharmacological activities, notably anti-inflammatory and antioxidant effects (Li et al., 2021; Gao et al., 2022; Yang et al., 2022). In rat models of PAH, oridonin has been shown to attenuate and even reverse disease progression by concomitantly modulating the nuclear factor-κB (NF-κB) and extracellular signal-regulated kinase (ERK) signaling pathways, thereby effectively suppressing inflammatory responses and alleviating oxidative stress (Wang et al., 2009).

#### 3.2.4 Triterpenoids

##### 3.2.4.1 Ginsenoside Rb1

Ginsenoside Rb1, a major protopanaxadiol-type saponin predominantly distributed in the roots of *Panax ginseng* C.A. Mey. and *Panax notoginseng* (Burkill) F.H. Chen ex C.Y. Wu and K.M. Feng (Fam. Araliaceae), exerts diverse pharmacological activities, including anti-inflammatory, antioxidant, anti-fibrotic, and cardiovascular protective effects (Gong et al., 2022; Liu J. et al., 2022). In the context of PAH, ginsenoside Rb1 has been shown to effectively lower PVR by inhibiting the activity of store-operated calcium entry (SOCE) channels, thereby reducing excessive pulmonary vasoconstriction (Wang R.-X. et al., 2015; Wang R.-X. et al., 2020).

##### 3.2.4.2 Ginsenoside Rg1

Ginsenoside Rg1, a major protopanaxatriol-type saponin predominantly derived from *Panax ginseng* and *Panax notoginseng*, exhibits multiple protective effects on the pulmonary vasculature (Wu J.-J. et al., 2024). In the context of PAH, ginsenoside Rg1 attenuates vascular inflammatory responses by downregulating the senescence-associated markers cyclin-dependent kinase inhibitor 1A (p21) and cyclin-dependent kinase inhibitor 2A (p16), thereby suppressing the cyclic GMP-AMP synthase/stimulator of interferon genes (cGAS/STING) signaling pathway (Ding et al., 2025). In addition, ginsenoside Rg1 upregulates the expression of cellular communication network factor 1 (CCN1), which in turn inhibits the TGF-β1/p-Smad2/3 signaling axis, effectively reversing hypoxia-induced endothelial dysfunction (Tang B.-L. et al., 2023). At the smooth muscle cell level, it suppresses the calpain pathway, thereby ameliorating abnormal hypoxia-driven proliferation of PASMCs (Ran et al., 2024). Furthermore, ginsenoside Rg1 reduces ROS generation and intracellular Ca^2+^ concentration in human pulmonary microvascular endothelial cells, while enhancing mitochondrial function and energy metabolism, thus preserving endothelial homeostasis (Chen et al., 2024).

##### 3.2.4.3 Ginsenoside Compound K

Ginsenoside Compound K, a rare secondary metabolite generated from protopanaxadiol-type ginsenosides via intestinal microbiota-mediated biotransformation, exhibits anti-inflammatory and vasoprotective properties (Jiao and Jia, 2022; Wang X. et al., 2022). In the context of PAH, ginsenoside Compound K has been shown to effectively inhibit PDGF-BB-induced aberrant proliferation of PASMCs, promote their apoptosis, and reverse pathological phenotypic switching. Through these combined actions, it intervenes at multiple mechanistic levels in the process of pulmonary vascular remodeling (Liu et al., 2021).

##### 3.2.4.4 Pachymic acid

Pachymic acid, a lanostane-type triterpenoid and secondary metabolite isolated from the medicinal fungus *Wolfiporia cocos* (F.A. Wolf) Ryvarden and Gilb. (syn. *Poria cocos*; Fam. Polyporaceae), possesses notable anti-oxidative stress and anti-proliferative effects on vascular smooth muscle cells (Zhu et al., 2021; He et al., 2022). In experimental models of PAH, pachymic acid has been shown to activate the Nrf2/Kelch-like ECH-associated protein 1 (Keap1)/antioxidant response element (ARE) signaling pathway, thereby mitigating oxidative damage, suppressing pathological cell proliferation, and ultimately ameliorating PAH-associated pulmonary vascular remodeling (He et al., 2022).

##### 3.2.4.5 Corosolic acid

Corosolic acid, a naturally occurring pentacyclic triterpenoid isolated from the leaves of *Lagerstroemia speciosa* (L.) Pers. (Fam. Lythraceae), exhibits potent vasoprotective potential in PAH. Experimental studies have shown that corosolic acid markedly suppresses the aberrant proliferation of PASMCs by inhibiting activation of the STAT3 signaling pathway (Kawade et al., 2023). In addition, it attenuates NF-κB activity, thereby downregulating PDGF receptor-β expression while concurrently inhibiting STAT3 expression and phosphorylation. Through these combined actions, corosolic acid effectively ameliorates PAH and its associated pathological vascular remodeling (Yamamura et al., 2024).

##### 3.2.4.6 Asiaticoside

Asiaticoside, a natural pentacyclic triterpenoid saponin predominantly obtained from the phytomedicine *Centella asiatica* (L.) Urb. (Fam. Apiaceae), possesses anti-inflammatory and anti-apoptotic properties (Dang et al., 2019; Yao et al., 2023). In the context of PAH, asiaticoside has been shown to activate the phosphatidylinositol 3-kinase (PI3K)/AKT/endothelial nitric oxide synthase (eNOS) signaling pathway, thereby enhancing nitric oxide (NO) production, suppressing endothelial cell apoptosis, and improving endothelial function (Wang et al., 2018a). Moreover, asiaticoside attenuates pulmonary vascular remodeling by inhibiting excessive activation of the TGF-β1/mothers against decapentaplegic homolog 2/3 (Smad2/3) signaling cascade, which in turn reduces the aberrant proliferation and migration of PASMCs while promoting their apoptosis (Wang X.-B. et al., 2015).

##### 3.2.4.7 18β-glycyrrhetinic acid

18β-Glycyrrhetinic acid, a principal bioactive triterpenoid aglycone predominantly isolated from the roots of *Glycyrrhiza glabra* L. (Fam. Fabaceae), exhibits antioxidant and anti-inflammatory effects, and has been shown to markedly improve pulmonary vascular function (Yang T. et al., 2021; Qing et al., 2022; Shanahati et al., 2025). In experimental models of PAH, 18β-glycyrrhetinic acid attenuates inflammation-mediated lung tissue injury, thereby slowing disease progression (Zhang et al., 2019b). Mechanistic studies indicate that 18β-glycyrrhetinic acid suppresses activation of the protein kinase RNA-like endoplasmic reticulum kinase (PERK)/eukaryotic translation initiation factor 2-alpha (eIF2α)/NF-κB signaling pathway, thereby mitigating endoplasmic reticulum stress (ERS)-induced inflammatory responses and alleviating pulmonary vascular remodeling (Wang J.-L. et al., 2022). Additionally, it modulates Ras homolog family member A (RhoA)/Rho-associated coiled-coil containing protein kinase (ROCK) signaling activity, which inhibits abnormal proliferation of human PASMCs (Zhang et al., 2019a).

##### 3.2.4.8 Ursolic acid

Ursolic acid, a naturally occurring pentacyclic triterpenoid widely distributed in plants such as *Malus domestica* Borkh. (apple), *Crataegus* spp. (hawthorn), and *Origanum vulgare* L. (oregano), possesses anti-inflammatory and anti-oxidative stress properties (Erdmann et al., 2021; Zhao et al., 2023). In animal models, ursolic acid has been shown to activate the peroxisome proliferator-activated receptor-α (PPARα)-dependent fatty acid oxidation pathway, thereby ameliorating RV energy metabolic disorders and subsequently suppressing the development and progression of myocardial hypertrophy, fibrosis, apoptosis, and functional impairment (Gao et al., 2020).

##### 3.2.4.9 Astragaloside IV

Astragaloside IV (ASIV), a principal cycloartane-type triterpene saponin isolated from the root of *Astragalus membranaceus* (Fisch.) Bunge (Fam. Fabaceae), has long been employed in traditional Chinese medicine for the prevention and treatment of cardiovascular diseases (Yang et al., 2023). It exhibits pronounced anti-inflammatory, anti-oxidative, and cardioprotective activities (Zhang D.-Q. et al., 2018). In experimental models of PAH, ASIV significantly lowers PAP, attenuates RVH, and alleviates pulmonary vascular remodeling, thereby preserving lung structural integrity and functional stability (Zhang X. et al., 2018; XinTian et al., 2022). Mechanistic studies have revealed that ASIV mitigates disease progression by downregulating TGF-β and IL-6 levels, consequently suppressing pro-inflammatory signaling cascades (Jin et al., 2021; Niu et al., 2025). Moreover, ASIV enhances endothelial function via upregulation of silent information regulator 1 (SIRT1), which reduces oxidative stress and limits endothelial injury (Leng et al., 2018; Zhang et al., 2021; Niu et al., 2025). It also modulates the Notch signaling pathway-closely implicated in PASMCs proliferation and apoptosis-whereby its inhibition under hypoxic conditions markedly attenuates pulmonary vascular remodeling (Yao et al., 2021). At the vascular protection level, ASIV improves endothelium-dependent vasodilation through SIRT1 upregulation and histone deacetylase (HDAC) inhibition, leading to enhanced eNOS expression and ultimately amelioration of PAH (Leng et al., 2018; Zhang et al., 2021).

##### 3.2.4.10 Celastrol

Celastrol, a bioactive pentacyclic triterpenoid isolated from the phytomedicine *Tripterygium wilfordii* Hook. f. (Fam. Celastraceae), possesses a broad spectrum of pharmacological activities, with anti-inflammatory and anti-oxidative effects being particularly prominent (Li Z. et al., 2022; Tan et al., 2025). In experimental models of PAH, celastrol markedly reduces PAP and mitigates medial hypertrophy as well as interstitial thickening of small pulmonary arteries (Li et al., 2022a). At the cellular level, under hypoxic conditions, celastrol directly suppresses the proliferation of human PASMCs by downregulating the pro-proliferative basigin (Bsg)/cyclophilin A (CyPA) signaling axis, decreasing intracellular ROS production, and reprogramming mitochondrial energy metabolism, thereby collectively inhibiting abnormal PASMCs growth (Kurosawa et al., 2019; 2021). Furthermore, in lung tissues, celastrol significantly attenuates inflammatory responses by inhibiting NF-κB signaling, contributing to its protective effects against pulmonary vascular remodeling (Li et al., 2022a).

## 4 Molecular mechanisms underlying the therapeutic effects of terpenoids on PAH

**FIGURE 3:**
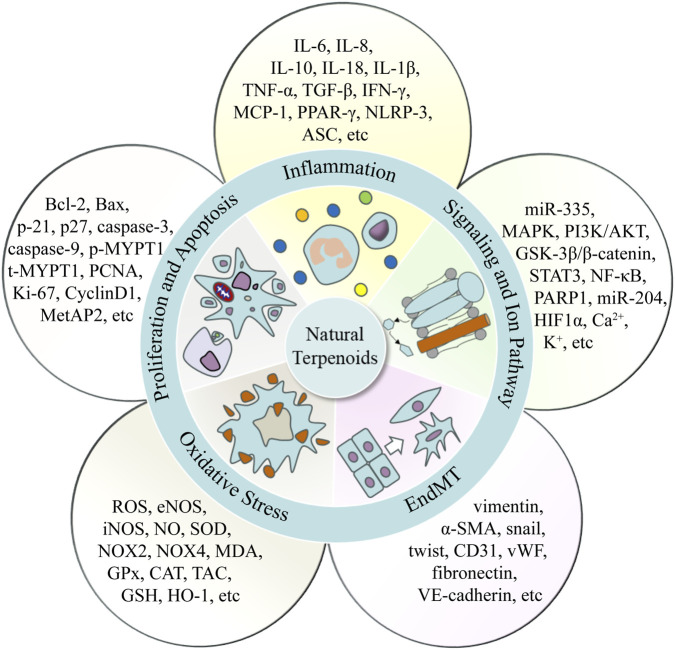
Regulation of PAH by natural terpenoids, including oxidative stress, inflammation, EndMT, proliferation and apoptosis, and ion channel activity.

As illustrated in Figure 3, natural terpenoids exert multifaceted, multi-target effects on modulating the development and progression of PAH. Their underlying mechanisms encompass the attenuation of oxidative stress, suppression of inflammatory responses, inhibition of EndMT, regulation of cellular proliferation and apoptosis, and modulation of ion channel activity.

### 4.1 Attenuation of oxidative stress

PAH can stimulate the excessive production of ROS in PASMCs, triggering oxidative stress and disrupting the normal function of multiple cellular signaling pathways. Oxidative stress may serve either as the initiating factor or as a downstream consequence of in various pathological processes, including hypoxia, inflammatory responses, and DNA damage, which in turn lead to an imbalance between PASMCs proliferation and apoptosis, pulmonary vascular remodeling and luminal narrowing, as well as abnormal alterations in vascular structure—ultimately contributing to the onset and progression of PAH (Liu H. et al., 2023; Tang et al., 2025). Therefore, suppression of oxidative stress and elimination of excessive ROS are considered effective strategies for the prevention and treatment of PAH.

#### 4.1.1 Direct modulation of oxidative stress factors

The accumulation of ROS is intimately linked to the disruption of redox homeostasis and mitochondrial function. During the onset and progression of PAH, excessive ROS generation drives oxidative stress, pulmonary vascular remodeling, and abnormal proliferation of PASMCs. Natural terpenoids confer protective effects by scavenging ROS, alleviating oxidative stress, and promoting PASMCs apoptosis, thereby attenuating pulmonary vascular remodeling and reducing elevated PAP. In high-altitude-induced PAH animal models, tanshinone IIA has demonstrated a pronounced protective effect against hypoxia. In mice exposed to acute hypobaric hypoxia at an altitude of approximately 4,260 m, tanshinone IIA treatment markedly prolonged survival time by increasing the activities of SOD and LDH and reducing MDA levels in both brain and cardiac tissues after hypoxia exposure (Wang et al., 2020b). In an *in vitro* PASMCs model exposed to 24 h of hypoxia (3% O_2_), carvacrol markedly decreased MDA levels and restored hypoxia-depleted SOD activity and reduced glutathione (GSH) content, thus attenuating oxidative stress (Zhang et al., 2016).

#### 4.1.2 Attenuation of aberrant pulmonary vasoconstriction

Oxidative stress can precipitate endothelial dysfunction and disrupt the finely tuned balance between vasoconstrictors and vasodilators in the pulmonary vasculature. Mechanistically, excessive oxidative stress inhibits eNOS activity and reduces NO bioavailability, thereby impairing endothelium-dependent vasodilation, inducing abnormal pulmonary vasoconstriction, and facilitating vascular remodeling. Consequently, restoration of vasodilatory capacity through antioxidant interventions represents a rational strategy to re-establish normal pulmonary vascular tone and attenuate PAH (Bao et al., 2023; Yu et al., 2025).

Experimental studies have demonstrated that tanshinone IIA can prevent the development of PAH in rats exposed to hypobaric hypoxia at an altitude of approximately 7,620 m (25,000 ft). Pretreatment with tanshinone IIA prior to each hypoxic exposure effectively inhibited hypoxia-induced ROS accumulation and lipid peroxidation in lung tissue, while increasing the GSH/oxidized glutathione (GSSG) ratio, SOD activity, and the expression of Nrf2, heme oxygenase-1 (HO-1), and metallothionein (MT). These changes enhanced the endogenous antioxidant defense capacity of hypoxic lung tissue and reduced oxidative injury. Given that NO and endothelin-1 (ET-1) are major vasoactive mediators governing vasodilation and vasoconstriction, respectively, tanshinone IIA pretreatment not only significantly increased the levels of NO, inducible nitric oxide synthase (iNOS), Na^+^/K^+^-ATPase, and vascular endothelial growth factor (VEGF), but also decreased ET-1 expression. This dual modulation reduced pulmonary vascular contractility and permeability, suppressed pathological vasospasm, and ameliorated vascular abnormalities. Collectively, by reinforcing the antioxidant defense system, rebalancing the NO/ET-1 axis, and improving vasomotor function, tanshinone IIA pretreatment effectively protected against hypoxia-induced pulmonary vascular injury and mitigated PAH progression (Yadav et al., 2025).

#### 4.1.3 Suppression of positive feedback loops associated with inflammation

During the development and progression of PAH, oxidative stress induces the excessive accumulation of reactive oxygen species (ROS). This accumulation directly activates inflammatory-related signaling pathways, and promotes the release of pro-inflammatory cytokines including IL-1β. Furthermore, it upregulates the expression of inflammation-mediated oxidases, which in turn increases ROS production, thus forming a vicious “oxidative stress - inflammation” positive feedback loop. Elevated ROS levels can promote phenotypic transformation and abnormal proliferation of PASMCs, directly leading to pulmonary vascular remodeling (Liu H. et al., 2023). The inflammatory environment can trigger endothelial dysfunction and vascular inflammation, thereby creating a self-perpetuating positive feedback loop between oxidative stress and inflammation. Over time, this vicious cycle accelerates pulmonary artery remodeling and leads to the occurrence and development of PAH (Poyatos et al., 2023).

In a MCT-induced PAH rat model, crocin markedly inhibited inflammatory signaling mediated by the chemokine CCL2 and its receptor CCR2. Treatment with crocin significantly downregulated both mRNA and protein expression of TNF-α, IL-6, IL-1β, and iNOS in lung tissue. Furthermore, oxidative stress biomarkers in PAH rat lungs showed marked dysregulation, with increased MDA levels and decreased activities of SOD and GSH-PX. Crocin intervention effectively reversed these alterations, thereby mitigating oxidative injury. Regarding RV remodeling, PAH model rats exhibited substantially elevated mRNA and protein levels of TNF-α, IL-6, and IL-1β in RV tissue, accompanied by cardiomyocyte hypertrophy, thickened myocardial fibers, interstitial inflammatory cell infiltration, and edema. Crocin treatment reduced the expression of these inflammatory mediators, restored normal myocardial architecture, and alleviated cardiac hypertrophy and inflammation-associated injury. Consequently, crocin ameliorated pulmonary vascular remodeling and partially prevented PAH progression. Collectively, these findings suggest that crocin exerts protective effects in PAH by simultaneously suppressing oxidative stress and inflammatory responses, thereby improving pathological alterations in both the pulmonary vasculature and cardiac tissue (Sheng et al., 2022).

In the same model, paclitaxel treatment also effectively reversed the reduction in endogenous antioxidant enzyme activities (such as SOD, GSH-Px, and CAT) and total antioxidant capacity (TAC), as well as the increase in malondialdehyde (MDA) levels. At the same time, it reduced the expression of pro-inflammatory cytokines TNF-α and IL-6 in lung tissue. Paclitaxel restored antioxidant enzyme activities and total antioxidant capacity and inhibited the production of pro-inflammatory cytokines. These molecular and biochemical improvements translated into better hemodynamic function, including reduced right ventricular systolic pressure and decreased pulmonary artery wall thickness, ultimately alleviating the severity of PAH (Beik et al., 2021). Further studies have shown that paclitaxel also downregulated the expression of microRNA-204 (miR-204) in the right ventricle of PAH rats, increased the antioxidant/oxidant ratio, and improved ventricular function, thereby exerting significant cardioprotective effects (Rajabi et al., 2021).

#### 4.1.4 Activation of the OXR1 signaling pathway

Oxidation resistance 1 (OXR1) is a critical regulatory factor in the cellular oxidative stress response. It mitigates oxidative damage primarily by upregulating the transcription of genes encoding antioxidant enzymes, thereby indirectly scavenging excessive ROS (Xu et al., 2023). Experimental evidence indicates that crocin can activate the OXR1 signaling pathway in PAH. In MCT-induced PAH rats, pulmonary tissue exhibited significantly reduced expression of OXR1 and its downstream target genes p21 and Nrf2 compared with control animals. Crocin intervention markedly reversed these changes, concomitantly activating the p21/Nrf2 antioxidant pathway. As a result, crocin restored the activities of GSH-Px, SOD, CAT, and GSH, while reducing MDA levels, thereby substantially enhancing overall antioxidant capacity. Beyond its antioxidative effects, crocin reduced collagen deposition in pulmonary tissue, decreased pulmonary arterial wall thickness, suppressed inflammatory cell infiltration, and improved histopathological architecture in the lungs of MCT-induced PAH rats. Collectively, these actions effectively inhibited the onset and progression of PAH. In summary, crocin exerts protective effects on the pulmonary vasculature and lung tissue by activating the OXR1/p21/Nrf2 antioxidant signaling axis, thereby synergistically attenuating oxidative stress and inflammatory responses (Xu et al., 2023).

#### 4.1.5 Regulation of the Nrf2/Keap1/ARE signaling pathway

**FIGURE 4:**
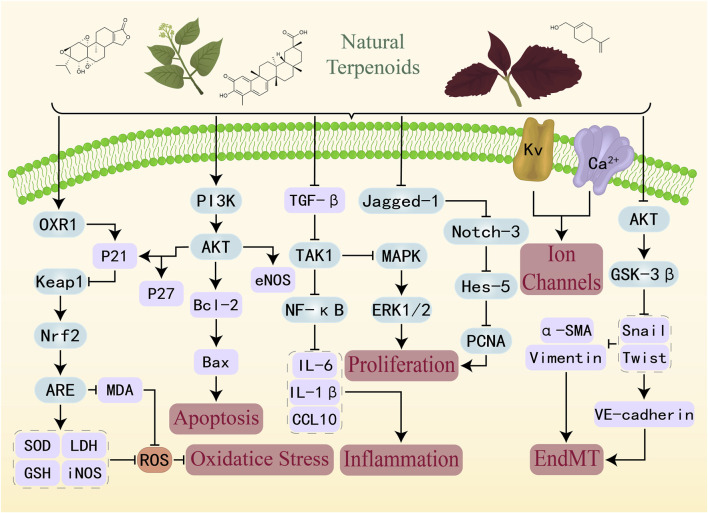
Molecular mechanisms of natural terpenoids in the treatment of PAH: inhibition of oxidative stress, suppression of inflammation, blockade of EndMT, regulation of cell proliferation and apoptosis, and regulation of Ion Channels.

Nrf2 is a stress-responsive transcription factor that plays a pivotal role in coordinating the cellular defense mechanisms against oxidative stress-induced injury. By regulating the transcription of a wide array of antioxidant and cytoprotective genes, Nrf2 helps maintain redox homeostasis and intracellular equilibrium (Hasan et al., 2025). Under basal conditions, Keap1 binds Nrf2 in the cytoplasm and promotes its ubiquitination and proteasomal degradation, thereby keeping intracellular Nrf2 levels low. Upon oxidative stress, conformational modification of Keap1 leads to its dissociation from Nrf2, allowing Nrf2 to translocate into the nucleus, bind to ARE in target gene promoters, and initiate transcription of downstream antioxidant genes (Tossetta et al., 2023). As shown in the Nrf2 part in Figure 4.

Preclinical studies have demonstrated that pachymic acid can alleviate hypoxia-induced PAH in rats via activation of the Nrf2/Keap1/ARE signaling pathway, thereby reversing RVH and pulmonary vascular remodeling (He et al., 2022). *In vivo*, administration of pachymic acid significantly decreased MDA levels in lung tissue, while upregulating the expression and activity of GSH-Px and SOD. *In vitro* experiments using hypoxia-exposed PASMCs further confirmed that hypoxic stress downregulated Nrf2 expression and upregulated Keap1 expression, whereas pachymic acid treatment effectively reversed these molecular alterations. Notably, co-administration of an Nrf2 inhibitor abolished the protective effects of pachymic acid, indicating that its anti-PAH efficacy is critically dependent on Nrf2/Keap1/ARE pathway activation. In summary, pachymic acid confers significant protection against hypoxia-induced PAH by modulating the Nrf2/Keap1/ARE axis, enhancing antioxidant defenses, and mitigating pulmonary vascular and cardiac remodeling.

### 4.2 Inhibition of inflammatory responses

Inflammation constitutes a pivotal pathological mechanism underlying the initiation and progression of PAH. In lung tissues from PAH rat models, pronounced neutrophil infiltration has been documented (Fadón-Padilla et al., 2024). The ensuing inflammatory microenvironment precipitates a cascade of pathological alterations in the pulmonary vasculature, including endothelial dysfunction, aberrant proliferation of PASMCs, and adventitial fibrosis. Collectively, these changes contribute to pulmonary vascular remodeling and an increase in PVR. Terpenoids exhibit significant cardiopulmonary protective potential by attenuating inflammatory responses and mitigating inflammation-mediated vascular pathology. Through suppression of pro-inflammatory signaling and downstream effector molecules, these compounds can effectively delay or prevent the structural and functional deterioration characteristic of PAH, thereby curbing disease development and progression.

#### 4.2.1 Regulation of pro-inflammatory cytokine release

Inflammatory processes can stimulate the release of a wide spectrum of pro-inflammatory cytokines. In patients with PAH, elevated levels of cytokines, inflammatory mediators, and immune cell infiltration are frequently observed around the pulmonary vasculature. Acting through multiple interconnected signaling pathways, these factors synergistically regulate pulmonary vascular remodeling and fuel the progression of PAH (Hu et al., 2020).

In a MCT-induced PAH rat model, D-limonene markedly attenuated cardiac inflammation and fibrosis, leading to improved cardiac function. PAH model rats exhibited significantly elevated mRNA expression of IL-1β, IL-6, and TNF-α in the right ventricle, alongside decreased levels of the anti-inflammatory cytokines IL-10 and TGF-β. Treatment with D-limonene restored these parameters to near-control levels, suggesting that its cardioprotective effects are primarily mediated by suppression of pro-inflammatory cytokine release, thereby alleviating myocardial inflammation and fibrosis and mitigating PAH severity (Teixeira-Fonseca et al., 2024).

Similarly, in MCT-induced PAH models, perillyl alcohol treatment effectively reduced RVSP and RVH index, normalized pulmonary artery wall thickness, and markedly improved pulmonary hemodynamics. Histopathological examination revealed severe pulmonary inflammatory responses in untreated PAH rats, characterized by elevated IL-1β and interleukin-8 (IL-8) levels, whereas perillyl alcohol administration significantly suppressed these inflammatory markers. These findings underscore the role of perillyl alcohol in suppressing pulmonary vascular inflammation, attenuating vascular remodeling, and alleviating PAH pathophysiology (Rajabi et al., 2020).

#### 4.2.2 Inhibition of the NF-κB signaling pathway

NF-κB is a key transcription factor regulating inflammatory responses. It can be activated via upstream Toll-like receptors (TLRs) or ROS signaling, and participates in the pathogenesis of various pulmonary diseases (Cui et al., 2025). In the pulmonary arterial intima of patients with chronic thromboembolic pulmonary hypertension, positive expression of phosphorylated NF-κB-p65 has been detected, indicating that the lungs are in an inflammatory state, with part of the process mediated by the NF-κB signaling pathway (Smolders et al., 2021). Activation of NF-κB promotes the expression of various pro-inflammatory mediators, such as IL-6 and chemokine CXCL10, both of which are significantly upregulated in PAH patients and experimental models (Wang R.-R. et al., 2022).

In a MCT-induced PAH rat model, prophylactic administration of paeoniflorin markedly reduced inflammatory cell infiltration in lung tissue, decreased the number of macrophages/monocytes and mast cells around pulmonary arteries, and downregulated mRNA expression of IL-6, IL-1β, and TNF-α. Furthermore, in PDGF-BB-induced PASMCs, paeoniflorin dose-dependently inhibited phosphorylation of the NF-κB p65 subunit, indicating that its anti-inflammatory effect is mediated, at least in part, through suppression of the NF-κB pathway, thereby preventing and treating PAH (Yu M. et al., 2022).

In addition, in a PDGF-BB-induced human PASMCs model, 18β-glycyrrhetinic acid inhibited endoplasmic reticulum stress activation, reduced phosphorylated NF-κB p65 expression, and upregulated IκB protein levels, thereby preventing NF-κB nuclear translocation. Animal experiments demonstrated that this compound significantly improved hemodynamic and histopathological parameters, reduced the RVH index, and alleviated pulmonary vascular remodeling. These findings suggest that the therapeutic effect of 18β-glycyrrhetinic acid on PAH is likely mediated through inhibition of the NF-κB signaling pathway (Wang J.-L. et al., 2022). As shown in the NF-κB part of Figure 4.

### 4.3 Inhibition of EndMT

EndMT is a pathological process in which PAECs lose their characteristic endothelial phenotype and functions, concomitantly acquiring mesenchymal-like properties such as enhanced migratory and invasive capacities. This phenotypic shift is marked by downregulation of endothelial markers [e.g., vascular endothelial cadherin, cluster of differentiation 31 (CD31)] and upregulation of mesenchymal markers (e.g., α-SMA, vimentin). EndMT plays a pivotal role in pulmonary arterial remodeling, contributing to medial and adventitial thickening, luminal narrowing, and a sustained increase in PAP. Over time, these structural and hemodynamic alterations drive both the onset and progression of PAH (Jiao et al., 2025).

#### 4.3.1 Activation of the BMP pathway

Loss-of-function mutations in the BMPR2 gene are among the leading genetic causes of both familial and idiopathic PAH. Such mutations attenuate canonical bone morphogenetic protein (BMP) signaling while relatively enhancing TGF-β signaling, disrupting the homeostatic balance between these two pathways. This imbalance facilitates EndMT, promotes vascular remodeling and luminal narrowing of pulmonary arteries, and ultimately contributes to PAH onset and progression (Sharmin et al., 2021; Wang C. et al., 2022).

In a SU5416/hypoxia (SuHx)-induced PAH rat model, prophylactic administration of paeoniflorin markedly attenuated RVH and fibrosis. Lung tissue from SuHx-exposed rats displayed significant downregulation of the endothelial marker vascular endothelial cadherin (VE-cadherin) and concomitant upregulation of mesenchymal markers, including fibronectin and vimentin—hallmarks of EndMT. Paeoniflorin treatment restored VE-cadherin expression and reduced fibronectin and vimentin levels, suggesting that it suppresses EndMT *in vivo*. Consistent results were observed in an *in vitro* hypoxia-induced human PAECs model, where hypoxia triggered EndMT in a time-dependent manner. This was characterized by marked upregulation of α-SMA, fibronectin, vimentin, and EndMT-associated transcription factors Snail and Twist, alongside reduced VE-cadherin expression. Paeoniflorin reversed the hypoxia-induced downregulation of VE-cadherin and partially attenuated the upregulation of vimentin and fibronectin, thereby mitigating EndMT progression. Mechanistic investigations revealed that hypoxia significantly decreased BMPR2 expression and phosphorylation of its downstream effectors SMAD1/5 in human PAECs, whereas paeoniflorin effectively restored both BMPR2 protein levels and SMAD1/5 phosphorylation. Importantly, siRNA-mediated BMPR2 knockdown markedly blunted paeoniflorin’s inhibitory effect on hypoxia-induced EndMT, indicating that its anti-EndMT activity is dependent on BMPR2 upregulation. In summary, paeoniflorin suppresses hypoxia- and SuHx-induced EndMT by restoring BMPR2 expression and activating the BMPR2–SMAD1/5 signaling axis, thereby attenuating pulmonary vascular remodeling and contributing to the prevention and treatment of PAH (Yu et al., 2020).

#### 4.3.2 Inhibition of the AKT/GSK-3β signaling pathway

The AKT signaling pathway can phosphorylate and inhibit glycogen synthase kinase-3β (GSK-3β), thereby preventing the proteasomal degradation of β-catenin. Aberrant accumulation of β-catenin is a critical driver of EndMT and can potentiate pulmonary vascular remodeling (Wu et al., 2023).

In a MCT-induced PAH rat model, curcumol exhibited dose-dependent protective effects, significantly reducing RVH and pulmonary arterial wall thickening. Lung tissues from MCT-treated rats displayed markedly elevated protein levels of phosphorylated AKT (p-AKT) and phosphorylated GSK-3β (p-GSK-3β), indicating activation of the AKT/GSK-3β cascade. Curcumol administration notably suppressed this phosphorylation, concomitant with downregulation of overexpressed mesenchymal markers—vimentin and α-SMA—as well as the EndMT-associated transcription factor Snail, thereby reversing MCT-induced EndMT *in vivo*. Consistent findings were observed *in vitro*. Rat PAECs exposed to EndMT-inducing medium exhibited a significant increase in the proportion of α-SMA-positive cells, decreased protein expression of endothelial markers CD31 and von Willebrand factor (vWF), and elevated vimentin, α-SMA, and Snail expression. Curcumol treatment reversed these alterations, restoring CD31 and vWF levels while reducing mesenchymal marker and transcription factor abundance. Moreover, curcumol inhibited p-AKT and p-GSK-3β expression in PAECs. Notably, co-administration of curcumol with the PI3K inhibitor LY294002 resulted in lower vWF protein expression than LY294002 treatment alone, indicating that curcumol’s modulation of EndMT is mechanistically linked to suppression of the AKT/GSK-3β axis rather than redundant with PI3K inhibition. In summary, curcumol attenuates the initiation and progression of EndMT by inhibiting the AKT/GSK-3β/β-catenin signaling pathway, thereby reducing vascular remodeling and improving cardiopulmonary function in PAH (Nie et al., 2023). As shown in the AKT part of Figure 4.

### 4.4 Regulation of proliferation and apoptosis

Abnormal proliferation of PASMCs is a central pathological feature of PAH, driving progressive pulmonary vascular wall remodeling and luminal narrowing. In this context, PASMCs frequently acquire apoptosis resistance, thereby enhancing their survival and propensity for accumulation (Vitry et al., 2021). In parallel, excessive apoptosis of vascular endothelial cells (ECs)—coupled with dysfunction of residual ECs—constitutes another critical factor in vascular remodeling, further accelerating the initiation and progression of PAH (Humbert et al., 2004; Zhang Q. et al., 2024). This dual cellular imbalance—hyperproliferation of PASMCs combined with excessive apoptosis of ECs—represents a defining hallmark of vascular pathology in PAH, leading to sustained structural and functional compromise of the pulmonary vasculature. Consequently, therapeutic strategies that simultaneously suppress aberrant PASMCs proliferation and protect ECs from apoptosis are of particular significance. Natural terpenoids are increasingly recognized for exerting anti-PAH effects, at least in part, through restoring the proliferation–apoptosis equilibrium within the pulmonary vascular wall.

#### 4.4.1 Regulation of the PI3K/AKT pathway

Activation of the PI3K/AKT signaling pathway can inhibit autophagy and apoptosis in vascular ECs, promoting a shift toward an anti-apoptotic phenotype (Tang Q. et al., 2023). In a hypoxia-induced PAH rat model, asiaticoside effectively suppressed pulmonary vascular remodeling and alleviated endothelial injury, accompanied by a marked upregulation of p-AKT/AKT and p-eNOS/eNOS in lung tissues. Similarly, in in vitro hypoxia-stimulated human PAECs, terminal deoxynucleotidyl transferase dUTP nick-end labeling assay and caspase-3 activity measurements showed that hypoxia significantly increased endothelial apoptosis, whereas asiaticoside treatment substantially reversed this effect, reducing both apoptotic cell proportion and caspase-3 enzymatic activity. Mechanistic analyses demonstrated that asiaticoside markedly enhanced the phosphorylation levels of AKT and eNOS under hypoxic conditions. Importantly, this effect was abolished by co-treatment with the PI3K inhibitor LY294002, indicating that asiaticoside’s anti-apoptotic and vasculoprotective activity is dependent on activation of the PI3K/AKT signaling cascade. In summary, asiaticoside mitigates apoptosis of human PAECs by activating the PI3K/AKT/eNOS pathway, thereby reducing endothelial injury, alleviating pulmonary vascular remodeling, and improving the pathological profile of hypoxia-induced PAH (Wang et al., 2018a). As shown in the PI3K part of Figure 4.

#### 4.4.2 Inhibition of the MAPK pathway

The MAPK signaling pathway is one of the central molecular mechanisms driving the onset and progression of PAH, comprising major subfamilies such as ERK1/2, c-Jun N-terminal kinase, and p38 MAPK. Hypoxia, inflammatory cytokines, and diverse growth factors can activate the MAPK cascade, leading to induction of downstream transcription factors and cell cycle regulators (e.g., Cyclins). This activation promotes the phenotypic switch of PASMCs from a quiescent, contractile phenotype to a synthetic state, characterized by abnormal proliferation, increased migratory capacity, and resistance to apoptosis—ultimately accelerating pulmonary vascular remodeling (Vanderpool et al., 2017).

In hypoxia-induced PASMCs, ASIV decreased the proportion of proliferating cell nuclear antigen (PCNA)-positive cells, downregulated hypoxia-induced expression of hypoxia-inducible factor-1α (HIF-1α) and phosphorylated ERK1/2, while upregulating cell cycle inhibitors p27 and p21 as well as pro-apoptotic proteins Bax, caspase-9, and caspase-3 (Jin et al., 2021).

It is noteworthy that the regulatory effects of ASIV on ERK1/2 phosphorylation appear to be context-dependent, varying with disease model and cell type. For instance, in certain endothelial injury models, ASIV has been reported to activate ERK1/2, thereby promoting endothelial repair rather than inhibition (Liu Y. et al., 2023). As shown in the MAPK/ERK part of Figure 4.

#### 4.4.3 Inhibition of the Notch pathway

In patients with PAH, the Notch ligand Jagged-1 is markedly overexpressed in PASMCs and drives their sustained proliferation through selective activation of Notch3 signaling in an autocrine manner. This aberrant activation not only elevates PVR and facilitates PAH onset, but also induces oxidative stress and endoplasmic reticulum stress, while inhibiting vascular cell apoptosis (Zhang Y. et al., 2022).

Evidence further indicates that in PASMCs derived from PAH patients, Notch3 mutations can enhance ROS production and suppress NO synthesis, thereby reducing apoptosis and maintaining a contractile phenotype (Morris et al., 2023). In a hypoxia-induced PAH rat model, PCNA levels were significantly elevated and the walls of small pulmonary arteries were notably thickened. ASIV treatment markedly alleviated these pathological changes, reducing pulmonary vascular remodeling. *In vitro*, hypoxia significantly promoted PASMCs proliferation and increased the proportion of cells in S and G_2_/M phases of the cell cycle, whereas ASIV inhibited excessive proliferation and decreased the percentage of cells in these proliferative phases. Mechanistic investigations revealed that ASIV suppressed the expression of Jagged-1, Notch-3, and the downstream transcription factor Hairy and Enhancer of Split-5. In summary, ASIV appears to ameliorate pulmonary vascular remodeling and slow or reverse PAH progression by inhibiting the Notch/Jagged-1 signaling pathway, thereby blocking aberrant PASMCs proliferation and cell cycle progression (Yao et al., 2021). As shown in the Jagged-1 part of Figure 4.

### 4.5 Regulation of ion channels

Dysfunction of potassium (K^+^) ion channels represents one of the core pathological features of PAH, whereas aberrant activation of Ca^2+^ ion channels serves as a major driving force for pulmonary vascular remodeling. Alterations in ion channel function—whether due to abnormal membrane potential regulation, disruption of intracellular Ca^2+^ homeostasis, or pathological mutations in channel-encoding genes—collectively contribute to sustained pulmonary vasoconstriction, progressive structural remodeling of the pulmonary vasculature, and eventual right ventricular failure (Masson et al., 2022; Villegas-Esguevillas et al., 2023).

#### 4.5.1 Enhancing voltage-dependent potassium channel activity

Voltage-dependent potassium (Kv) channels are among the principal determinants of resting membrane potential in PASMCs (Mondéjar-Parreño et al., 2021). Downregulation of Kv channel expression or attenuation of Kv currents leads to membrane depolarization of PASMCs, which in turn reduces apoptosis, promotes excessive proliferation, and ultimately drives pulmonary vascular remodeling. Under acute hypoxic conditions, tanshinone IIA has been shown to reverse the hypoxia-induced suppression of Kv currents in PASMCs. In rat models of PAH induced by chronic intermittent or sustained hypoxia, tanshinone IIA treatment significantly lowered RVSP, reduced the RVH index, and attenuated pulmonary vascular remodeling. Furthermore, tanshinone IIA markedly upregulated both mRNA and protein expression of Kv1.5 and Kv2.1 channels in small pulmonary arteries, thereby restoring Kv currents in PASMCs. These findings suggest that tanshinone IIA may alleviate sustained pulmonary vasoconstriction and vascular structural remodeling by directly enhancing Kv channel expression and function, thus preventing membrane depolarization—an effect that may represent a key mechanism underlying its therapeutic efficacy in hypoxia-induced PAH (Zheng et al., 2015). As shown in the Kv part of Figure 4.

#### 4.5.2 Regulating intracellular Ca^2+^ concentration to inhibit vascular remodeling

Sustained elevation of intracellular Ca^2+^ concentration in PASMCs is a key driver of abnormal cell proliferation and migration, leading to vascular wall thickening and occlusive remodeling, and thus plays a central role in the pathogenesis of PAH (Masson et al., 2022; Zhang et al., 2024a). Modulation of voltage-dependent Ca^2+^ channel activity can directly influence intracellular Ca^2+^ homeostasis, thereby regulating pulmonary artery tone and overall hemodynamics (Jain et al., 2021).

In rat models of PAH induced by chronic hypoxia or MCT, ginsenoside Rb1 significantly inhibited pulmonary artery contraction induced by ET-1 and cyclopiazonic acid, and reduced both cyclopiazonic acid-activated Ca^2+^ influx and non-selective cation entry in PASMCs. Treatment with ginsenoside Rb1 markedly decreased the amplitude of cyclopiazonic acid-induced Ca^2+^ transients and the Mn^2+^ quenching rate, while restoring the elevated resting intracellular Ca^2+^ levels observed in PAH models. Importantly, the selective SOCE pathway inhibitor gadolinium completely abolished the vasodilatory effect of ginsenoside Rb1, indicating that its action is mediated through specific inhibition of the SOCE pathway. By lowering intracellular Ca^2+^ concentrations in PASMCs, ginsenoside Rb1 counteracts the augmented pulmonary vasoconstriction and hyperreactivity characteristic of PAH, thereby offering a potential therapeutic strategy grounded in Ca^2+^ signal modulation (Wang R.-X. et al., 2015). As shown in the Ca^2+^ part of Figure 4.

## 5 Safety considerations and therapeutic limitations

Although terpenoids demonstrate promising potential, their translation from basic research to clinical application is severely constrained by their toxicological profiles. The safety of these compounds varies significantly. They range from well-tolerated food additives to agents with narrow therapeutic indices that necessitate structural modification or targeted delivery systems.

### 5.1 Well-tolerated agents and dose-dependent effects

Many monoterpenes and specific terpenoids exhibit favorable safety profiles. This is supported by their historical use in traditional medicine and their status as food additives. Specifically, D-Limonene, 1,8-Cineole, and Carvacrol hold “Generally Recognized as Safe” status (Chen et al., 2016; Salehi et al., 2019). D-Limonene shows low toxicity. Nephropathy has been observed only in male rats, via a mechanism irrelevant to humans. However, high doses may cause gastrointestinal irritation (Kim et al., 2013). Similarly, 1,8-Cineole and Carvacrol are generally safe. Nevertheless, they may cause mucosal irritation. In cases of extreme overdose, they can lead to neurotoxicity (Khosravi and Erle, 2016; Dosoky and Setzer, 2018). Likewise, Crocin and Astragaloside IV show no significant cytotoxicity in major organs at effective pharmacological doses. They demonstrate high safety margins. This makes them ideal candidates for long-term PAH management (Ren et al., 2013; Alavizadeh and Hosseinzadeh, 2014). However, safety is not absolute. Perillyl alcohol is a structural analog of limonene. Yet, it exhibited dose-limiting gastrointestinal toxicity (nausea and vomiting) in clinical trials. This suggests that inhalation administration may be necessary for PAH treatment to bypass gastrointestinal adverse effects (DA Fonseca et al., 2013).

### 5.2 Compounds with narrow therapeutic windows

For certain diterpenoids and triterpenoids, the narrow margin between therapeutic efficacy and organ toxicity presents a major obstacle. Triptolide has a narrow therapeutic window. It exhibits multi-organ toxicity, particularly hepatotoxicity, nephrotoxicity, and severe reproductive toxicity (such as amenorrhea and inhibition of spermatogenesis). These issues strictly limit its systemic application (Xi et al., 2017). Celastrol is effective in remodeling pulmonary vasculature. However, it carries risks of cardiotoxicity and infertility. Furthermore, its poor water solubility often necessitates administration via toxic vehicles (Kannaiyan et al., 2011). Fumagillin is potent in inhibiting angiogenesis. Yet, its clinical development has historically been hindered by severe neurotoxicity and thrombocytopenia at therapeutic doses (Wei J. et al., 2022). Oridonin shows rapid plasma clearance in animal models. Additionally, it exhibits potential gastrointestinal toxicity (Yang Y.-S. et al., 2021).

### 5.3 Specific adverse effects relevant to PAH pathophysiology

Certain compounds may exhibit side effects that exacerbate cardiovascular instability in PAH patients. Long-term administration of 18β-Glycyrrhetinic acid induces pseudoaldosteronism. This results in sodium retention, hypokalemia, and hypertension (Isbrucker and Burdock, 2006). Fluid retention and systemic hypertension are particularly detrimental to PAH patients with compromised right ventricular function. Therefore, strict monitoring is required. Alternatively, the drug should be contraindicated in salt-sensitive individuals. Andrographolide typically exhibits hepatoprotective effects. However, cumulative high doses may lead to renal injury (Li X. et al., 2022).

### 5.4 Pharmacokinetic interactions

Tanshinone IIA, Ginsenoside Rb1, Ginsenoside Rg1, and Curcumol modulate CYP450 enzymes and P-glycoprotein (Qiu et al., 2014; Gao et al., 2021; Chen et al., 2022). Specifically, Tanshinone IIA may enhance or inhibit warfarin metabolism. This alteration increases the risk of bleeding. For PAH patients receiving anticoagulant therapy, this represents a critical safety consideration (Chen et al., 2019). Asiaticoside also displays varying degrees of CYP450 inhibitory potential. Therefore, rigorous interaction studies are essential before conducting clinical combination trials (Pan et al., 2014). In summary, agents like Astragaloside IV and D-Limonene appear ready for human safety testing. Conversely, compounds such as Triptolide, Celastrol, and Fumagillin may require nanoparticle encapsulation or structural modification to widen their therapeutic windows. Furthermore, caution is required in designing clinical trials for PAH. The mineralocorticoid-like effects of 18β-Glycyrrhetinic acid must be carefully managed. Similarly, the drug interaction potential of Ginsenosides and Tanshinone IIA requires strict attention.

## 6 Conclusions and perspectives

This systematic review identified 23 natural terpenoids encompassing diverse structural subtypes: 5 monoterpenes, 3 sesquiterpenes, 5 diterpenes, and 10 triterpenes. These compounds have been investigated primarily for their potential to modulate PAH-related signaling pathways and suppress aberrant proliferation of PASMCs. Accumulating evidence shows that natural terpenoids act through multiple, overlapping molecular mechanisms, including regulation of oxidative stress, suppression of inflammation, inhibition of EndMT, modulation of cell proliferation and apoptosis, and restoration of ion channel homeostasis. In terms of antioxidant effects, they regulate HIF-1α, OXR1, and Nrf2/Keap1/ARE signaling, lower MDA levels, and restore SOD and GSH activity. These actions enhance ROS clearance, alleviate oxidative stress–mediated PASMCs proliferation and apoptosis, and ultimately slow the progression of PAH (Wang et al., 2020b). To address the excessive PASMCs proliferation that constitutes a core pathological feature of PAH, terpenoids modulate PI3K/AKT, MAPK, and Notch pathways, and regulate Bcl-2 and Bax expression, thereby limiting PASMC growth and reducing apoptosis of human PAECs, which improves the proliferative/anti-apoptotic phenotype (Wang et al., 2018a).Anti-inflammatory effects are mediated through the suppression of IL-6 and CXCL10 via NF-κB signaling. They also involve the inhibition of NF-κB nuclear translocation. In some cases, these compounds downregulate endoplasmic reticulum stress activation. Collectively, these actions reshape the inflammatory microenvironment and slow PAH progression (Wang J.-L. et al., 2022). Regarding EndMT regulation, terpenoids influence the BMP and AKT/GSK3β pathways. This reduces EndMT activity and prevents endothelial phenotypic switching. Consequently, these effects delay vascular remodeling (Nie et al., 2023). Furthermore, these agents modulate ion channel dynamics by activating potassium channels to promote pulmonary vasodilation. They also inhibit calcium channel activation. This decreases Ca^2+^ influx and reduces vascular smooth muscle contractility. Ultimately, these combined mechanisms mitigate vascular remodeling (Wang R.-X. et al., 2015; Zheng et al., 2015). Overall, natural terpenoids display multi-target, multi-pathway, and pleiotropic therapeutic advantages in PAH, highlighting their promise as candidates for further drug development and clinical translation. The underlying mechanisms are summarized in Figure 4.

Natural terpenoids exhibit significant anti-inflammatory, anti-proliferative, and vasodilatory activities in preclinical PAH models. However, their clinical translation faces severe pharmacokinetic challenges. First, extremely low water solubility presents a primary barrier to absorption. Most terpenoids fall under Class II or IV of the Biopharmaceutics Classification System (BCS). Notable examples include Tanshinone IIA, Oridonin, Ursolic acid, Celastrol, Corosolic acid, and Pachymic acid. These compounds dissolve poorly in gastrointestinal fluids. Consequently, their oral bioavailability remains critically low (Rosa et al., 2020; Li M. et al., 2022; Li J. et al., 2022; Wei C. et al., 2022; Sobral et al., 2023; Kadsanit et al., 2024). Second, poor membrane permeability and efflux pump mechanisms further hinder systemic absorption. Macromolecular saponins, such as Astragaloside IV, Ginsenoside Rg1, and Asiaticoside, exhibit high polarity and molecular weight. As a result, they penetrate the intestinal epithelial barrier inefficiently (Han and Fang, 2006; Huang et al., 2006; Zhang and Zhang, 2015). Concurrently, Paeoniflorin and Andrographolide are confirmed substrates of P-glycoprotein. They undergo active efflux by intestinal epithelial cells, which significantly lowers plasma drug concentrations (Liu et al., 2006; Ye et al., 2011). Furthermore, poor metabolic stability and rapid elimination are key bottlenecks in maintaining effective therapeutic concentrations. Volatile monoterpenes (e.g., D-Limonene, Perillyl alcohol) and sesquiterpenes (e.g., Parthenolide, 1,8-Cineole) undergo extensive first-pass metabolism or oxidation *in vivo*. This results in extremely short half-lives (Cheng et al., 2005; McLean et al., 2007; Sun, 2007; Freund et al., 2020). In contrast, Crocin requires hydrolysis by intestinal flora into crocetin prior to absorption. This conversion process is inefficient and exhibits significant inter-individual variability (Zhang Y. et al., 2019; Shakya et al., 2020). Moreover, potent compounds like Triptolide and Fumagillin are associated with severe hepatorenal or neurotoxicity. Consequently, they possess narrow therapeutic windows (Stockmann-Juvala and Savolainen, 2008; Zhou et al., 2018). Given these multiple pharmacokinetic limitations, developing nanodelivery systems is essential to unlock the therapeutic potential of these natural products. Promising strategies include the use of liposomes, polymeric micelles, or solid lipid nanoparticles. These systems not only significantly improve solubility and bioavailability but also prolong circulation time. Additionally, they enable lung-targeted delivery, thereby enhancing efficacy while reducing systemic toxicity.

Despite promising preclinical prospects, most terpenoids have not entered registered clinical trials for PAH. Current evidence remains limited to *in vitro* studies and animal models. The primary bottlenecks impeding clinical application stem from unfavorable pharmacokinetic profiles. Specifically, these include extremely low oral bioavailability, non-specific tissue distribution, and narrow therapeutic indices. The dose-limiting toxicity of Triptolide serves as a notable example. To overcome these translational hurdles, future research must prioritize two strategies. The first involves optimizing physicochemical properties and metabolic stability via structural modification. The second entails developing advanced delivery systems, particularly inhalable nanocarriers. These systems enable targeted accumulation within the pulmonary vasculature, thereby reducing systemic toxicity. Simultaneously, a “drug repurposing” strategy should be adopted. Given their established safety profiles, small-scale exploratory clinical trials for PAH are warranted. Such efforts will facilitate the transition of these natural products from bench to bedside.

In conclusion, this review synthesizes the current knowledge regarding natural terpenoids in PAH treatment and underscores their considerable promise. These insights are expected to guide the development of next-generation therapeutic agents that are more effective, safer, and naturally derived, with substantial translational potential for clinical application.

## Glossary

Akt: Protein kinase B
ARE: Antioxidant response element
ASIV: Astragaloside IV
Bcl-2: B-cell lymphoma 2
BMP: Bone Morphogenetic Protein
BMPR2: Bone morphogenetic protein receptor type II
Bsg: Basigin
**Ca** ^**2+**^: Calcium
CAT: Catalase
CCL2: C-C motif chemokine ligand 2
CCN1: Cellular communication network factor 1
CCR2: C-C chemokine receptor 2
CD31: Cluster of differentiation 31
cGAS: Cyclic GMP–AMP synthase
CXCL1: C-X-C motif chemokine ligand 1
Cyclins: Cell cycle proteins
CyPA: Cyclophilin A
Ecs: Endothelial cells
eIF2α: Eukaryotic translation initiation factor 2-alpha
EndMT: Endothelial-to-mesenchymal transition
eNOS: Endothelial nitric oxide synthase
ERK: Extracellular signal-regulated kinase
ERS: Endoplasmic reticulum stress
ET-1: Endothelin-1
GSH: Glutathione
GSH-Px: Glutathione peroxidase
GSK-3β: Glycogen synthase kinase-3β
GSSG: Oxidized glutathione
HDAC: Histone deacetylase
HIF-1α: Hypoxia-inducible factor-1α
HO-1: Heme oxygenase-1
IL-10: Interleukin-10
IL-1β: Interleukin-1β
IL-6: Interleukin-6
IL-8: Interleukin-8
iNOS: Inducible nitric oxide synthase
**K** ^**+**^: Potassium
Keap1: Kelch-like ECH-associated protein 1
Kv: Voltage-dependent potassium
LDH: Lactate dehydrogenase
LPO: Lipid peroxides
MAPK: Mitogen-activated protein kinase
MCT: Monocrotaline
MDA: Malondialdehyde
MetAP2: Methionine aminopeptidase-2
miR-204: MicroRNA-204
MMP-2: Matrix metalloproteinase-2
MMP-9: Matrix metalloproteinase-9
Mpap: Mean pulmonary arterial pressure
MT: Metallothionein
NF-κB: Nuclear factor-κB
NO: Nitric oxide
Nrf2: Nuclear factor erythroid 2-related factor 2
OXR1: Oxidation resistance 1
p16: Cyclin-dependent kinase inhibitor 2A
p21: Cyclin-dependent kinase inhibitor 1A
PAECs: Pulmonary artery endothelial cells
PAH: Pulmonary arterial hypertension
p-AKT: Phosphorylated AKT
PAP: Pulmonary arterial pressure
PARP1: Poly(ADP-ribose) polymerase 1
PASMCs: Pulmonary arterial smooth muscle cells
PCNA: Proliferating cell nuclear antigen
PDGF: Platelet-derived growth factor
PERK: Protein kinase RNA-like endoplasmic reticulum kinase
p-GSK-3β: Phosphorylated GSK-3β
PI3K: Phosphatidylinositol 3-kinase
PPARα: Peroxisome proliferator-activated receptor-α
PVR: Pulmonary vascular resistance
RhoA: Ras homolog family member A
ROCK: Rho-associated coiled-coil containing protein kinase
ROS: Reactive oxygen species
RV: Right ventricular
RVH: Right ventricular hypertrophy
RVSP: Right ventricular systolic pressure
SIRT1: Silent information regulator 1
Smad2/3: Mothers against decapentaplegic homolog 2/3
SOCE: Store-operated calcium entry
SOD: Superoxide dismutase
STAT3: Signal transducer and activator of transcription 3
STING: Stimulator of interferon genes
SuHx: SU5416/hypoxia
TAC: Total antioxidant capacity
TGF-β1: Transforming growth factor-β1
TIMP-1: Tissue inhibitor of metal protease 1
TLRs: Toll-like receptors
TNF-α: Tumor necrosis factor-α
TRPM7: Transient receptor potential melastatin 7
VE-cadherin: Vascular endothelial cadherin
VEGF: vascular endothelial growth factor
Vwf: Von Willebrand factor
α-SMA: α-smooth muscle actin
Skp2: S-phase kinase-associated protein 2
